# Development and preliminary evaluation of a camera-assisted endovascular simulator using a three-dimensional printing

**DOI:** 10.1590/acb413826

**Published:** 2026-07-17

**Authors:** Bruno Tavares de Andrade, Carlo Átila Holanda Lopes, Yury Tavares de Lima, Victor Rocha Pincanço, Natan Rolim de Assunção Bisio, Henrique Luis do Carmo e Sa, Josué Viana de Castro

**Affiliations:** 1 Universidade de Fortaleza – Medical School – Surgical Skills and Simulation Center – Fortaleza (CE) – Brazil.

**Keywords:** Endovascular Procedures, Radiology, Interventional, Simulation Training, Patient Simulation

## Abstract

**Purpose:**

This study described the development and preliminary evaluation of a simulator that combines a three-dimensional-printed vascular model with a camera-assisted system for training basic endovascular skills.

**Methods:**

A hollow mannequin torso housed a transparent, three-dimensional-printed aorta. Guidewire and catheter manipulation were visualized in real time using a manually controlled camera. Fifteen medical students (novices) and five vascular surgeons (experts) performed simulated tasks in two sessions. Experts also assessed realism and educational value.

**Results:**

Novices demonstrated shorter completion times across all exercises in session 2, particularly in exercise 4, with mean time decreasing from 229.3 ± 134.8 to 83.5 ± 31.1 seconds, a mean difference of 145.8 seconds (*p* = 0.001). Experts showed no significant improvement in most tasks, although exercise 3 improved from 115.4 ± 29.7 to 91.6 ± 21.5 seconds, a mean difference of 23.8 seconds (*p* = 0.012). Performance differed between groups (construct validity). Surgeons provided favorable ratings for the realism and usefulness (face and content validity).

**Conclusion:**

This preliminary device appears feasible and may facilitate early skill acquisition in low-resource educational settings without radiation exposure.

## Introduction

Endovascular science has become a field of fast improvement in technology. Minimally invasive techniques have nowadays replaced open procedures in almost all vascular interventions. These continuous advancements have raised essential questions regarding optimizing surgical education and training to achieve proficiency goals^1,2^. Moreover, desirable safety standards may not be guaranteed, possibly resulting in legal implications, poor postoperative results, and economic impacts^3,4^.

Trainees tend to require more time to complete endovascular procedures, thus increasing the time of exposure to ionizing radiation for themselves, staff, and patients^5^. In addition, changes related to a reduced weekly workload or increased research activities significantly decreased the surgical volume^6,7^.

This scenario illustrates how the surgical educational system has been implemented over the years, compromising the autonomy and confidence demonstrated by many apprentices^8^. Simulation courses have become a fundamental tool for promoting learning and improving performance^9,10^. Realistic immersive simulators that allow multidisciplinary teamwork are the gold standard for initial training and skill acquisition in this field^11^.

Randomized controlled trials have demonstrated unequivocal superiority in performance improvement and shortening the learning curve using high-fidelity virtual reality simulators^12^. This is an excellent stimulus for regularly incorporating this instrument into Endovascular Surgery programs, but the investment required for acquisition and maintenance limits its use^13,14^.

Low-cost simulators appear to be viable alternatives in educational scenarios involving initial practice^15^. Despite the wide variety of studies reporting the usefulness of virtual reality-based simulators, only a few low-cost endovascular simulators with high functional fidelity have been described^16^. This pilot experiment promotes the development and preliminary evaluation of a simulator that offers a radiation-free environment and employs an image-guided method to train basic endovascular skills.

## Methods

### Study design

This prospective, single-center study was performed at the Center for Surgical Education at the Universidade de Fortaleza Medical School from May to June 2023. The project was submitted and approved by the Research Ethics Board of the Universidade de Fortaleza at Plataforma Brasil (number 67655823.1.0000.5052). This research was reported using the Strengthening the Reporting of Observational Studies in Epidemiology (STROBE) checklist with extensions for simulation-based research^17^.

### Ethical considerations

Research project was submitted to the Research Ethics Committee of the Universidade de Fortaleza and approved by it under registration number 6.042.409 and was conducted with the informed and appropriate consent of each participant, including authorization for audiovisual recording.

### Participants

Fifteen undergraduate medical students (novice group) and five vascular surgeons (expert group) were voluntarily recruited. Students were required to be actively enrolled in the Medicine program at the Universidade de Fortaleza and to have no prior specific training with endovascular simulators. Vascular surgeons were required to possess a minimum of three years of professional experience in the field, in addition to prior knowledge of and training with other endovascular simulators. We did not include vascular surgery residents because of their very limited numbers in our city.

Participants were excluded if they did not meet the predefined experience criteria for their respective group, declined participation at any stage of the study, were unable to complete the simulation protocol, or had incomplete performance data that prevented analysis. No enrolled participants met these exclusion criteria after inclusion.

### Simulator development and production

A plastic mannequin representing the human torso was prepared and fixed to a wooden board for stabilization. The main author drafted a representation of the aorta and its main branches. To define the diameters of the vessels represented in the final model, previously published templates were reviewed^18,19^, and population studies were analyzed to represent the arterial calibers found in healthy individuals obtained from computed tomography scans^20,21^.

**Figure 1:**
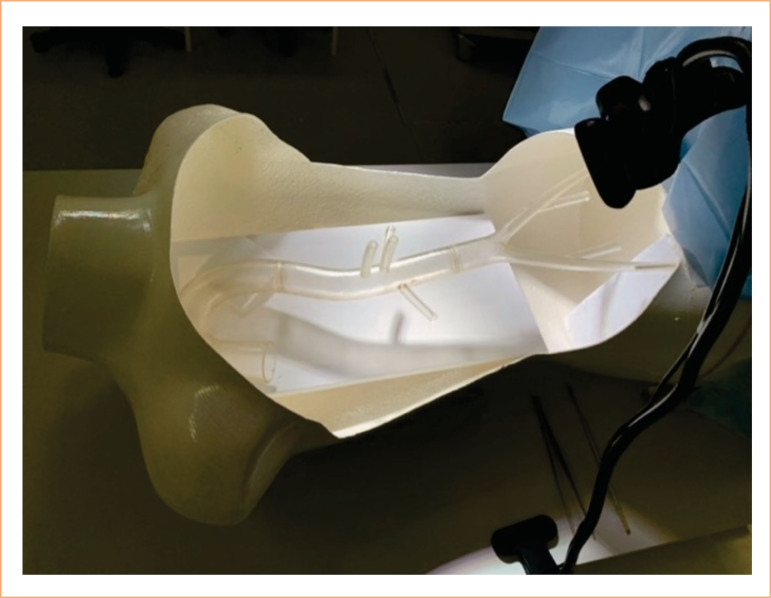
Final aspect of the endovascular simulator.

Source: Elaborated by the authors.

**Figure 2:**
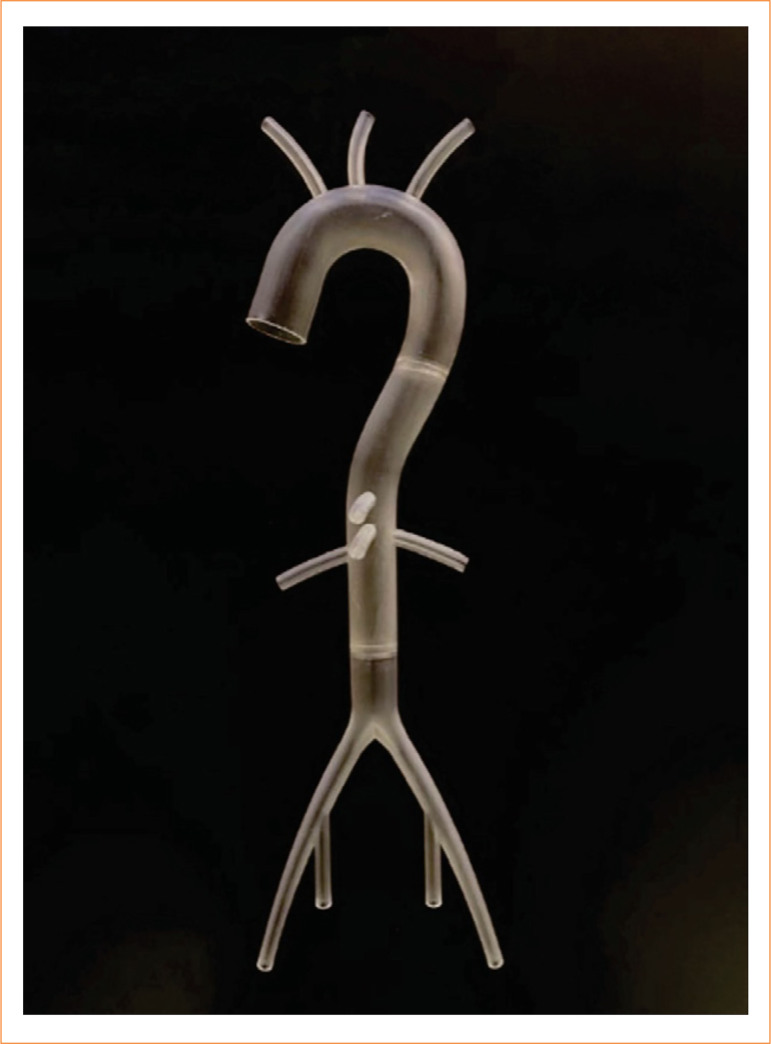
Final arterial model after three-dimensional printing.

Source: Elaborated by the authors.

The next stage consisted of three-dimensional modeling using CAD software (Autodesk, San Rafael, California, United States of America)^22^. An Anycubic Photon Mono X 4K three-dimensional printer (ANYCUBIC 3D Printing, Shenzhen, China) was used to produce the final model components. This device can print objects up to 15 centimeters long. Therefore, the model was divided into three parts that were printed separately and assembled into a single piece after curing. The three-dimensional Ink Geek^©^ resin VITRO color (3D Ink Resinas^©^, Contagem, MG, Brazil) provided good resistance and enabled the manufacture of transparent parts, enhancing the final visualization of the interior (Fig. 1). A layer of colorless glass varnish (Acrilex Tintas Artísticas©, São Paulo, SP, Brazil) was applied to the internal and external surfaces to maintain protection and transparency (Fig. 2).

Image capture was performed using a 3-megapixel resolution camera (Logitech International S.A., Lausanne, Switzerland) connected to a screen through a universal serial bus (USB) device. The camera remained suspended over the simulated surgical field using an articulated plastic support fixed with a clip to the edge of the wooden support (VEXclip, Shanghai, China).

### Procedure and data collection

Initially, each volunteer answered a demographic questionnaire. In a short session (maximum of 7 minutes), medical students were instructed about how the simulator worked, the instruments employed, and the procedural five steps:

- Identification and progression of a flexible J tip 0.035” hydrophilic guide wire to the thoracic aorta;
- Identification and progression of the Pigtail catheter over the 0.035” guide wire to the thoracic aorta;
- Exchanging the Pigtail catheter for a Cobra 2 catheter and perform selective catheterization of the left renal artery;
- Crossing the aortic bifurcation to the left iliac artery with a Cobra 2 catheter and selective catheterization of the left internal iliac artery;
- Exchanging the Cobra 2 catheter for a balloon catheter with its insufflation above the level of the celiac trunk.

The total time for each task was recorded, with no opportunity for repetition. After finishing the first cycle, students could freely practice previously performed skills for 30 minutes. None of the volunteers knew which simulation was to be performed at the time of the experiment.

The primary objective of this study was to evaluate the reduction in time required to complete the tasks following training.

The group of vascular surgeons was introduced to the simulator, available instruments, and tasks to be performed. Subsequently, the participants proceeded directly to the protocol. Each step was timed sequentially with no possibility of repetition, and the entire cycle was repeated once more. After the second cycle, they answered a questionnaire regarding their perception of the educational usefulness of the simulator in terms of content and face validity. The answers were evaluated using a Likert scale^23^, with scores ranging from 1 to 5 (1 = strongly disagree, 2 = disagree, 3 = neutral, 4 = agree, and 5 = strongly agree). Rates ranging from 1 (worst) to 10 (best) were used for realism (general configuration, guidewires and catheters manipulation, projected image and exercises performed)^24^.

All participants individually used the simulator with the help of the main researcher, who timed each exercise, and another assistant, who moved the camera, as requested by the volunteers. The operator handled the inguinal region of the mannequin in which the 12 Fr introducer sheath was correctly positioned to simulate a right femoral arterial puncture. Care was taken during the exercises to use surgical drapes to ensure that the volunteers handled the wires and catheters by seeing the real-time images on a screen and not under direct visualization of the field. The participants did not previously meet the research team; they were only allowed to ask for camera adjustments or to inform the beginning and end of each simulation stage for register. Any other form of communication during the procedure was avoided, and the sessions were recorded using the same video apparatus.

The location of the experiment was calm and silent, and access was restricted to the participants and researchers involved in the protocol. To mitigate measurement bias, the same two evaluators (the main and assistant researchers) participated in all tests. In case of disagreement between the two evaluators regarding the measured data, a third evaluator could intervene. All questions were answered, and both exercises were completed by all participants.

### Statistical analysis

Categorical variables are presented as absolute counts and relative frequencies in percentages. The χ^2^ test or Fisher’s exact test was used to assess the association between categorical variables, depending on the expected frequencies in the 2 × 2 tables. Continuous variables were initially evaluated for normality using the Shapiro-Wilk’s normality test, evaluation of Q-Q plots, and histograms. Normally distributed continuous variables were graphically reported as mean ± standard deviation or mean ± standard error. For normally distributed continuous variables, dependent comparisons between the two groups were performed using paired t-tests. Independent comparisons of means between the two groups were conducted using the Student’s t-test. Data were analyzed using Statistical Package for the Social Sciences software for Macintosh, version 23 (IBM Corp., Armonk, NY, United States of America). For all tests, a significance level of *p* < 0.05 was considered statistically significant.

## Results

**Figure 3:**
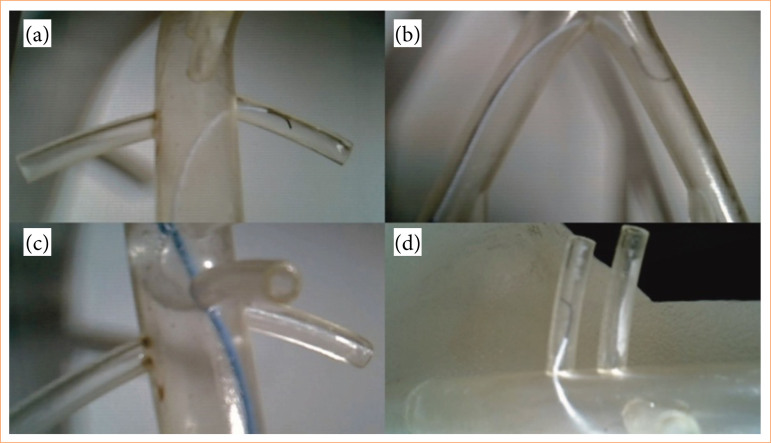
Projected images from the camera. (a) Left renal artery catheterization. (b) Aortic bifurcation crossover. (c) Balloon insufflation. (d) Celiac trunk catheterization.

Source: Elaborated by the authors.

Our simulator met the expectations of realism in terms of dimensions and proportions. The assembly process was uneventful, with the longest period dedicated to producing the three-dimensional printed transparent arterial model in resin. The final product exhibited a high degree of transparency, which enabled proper visualization of instrument navigation, resulting in a single hollow piece with constant thickness (Fig. 3). The camera allowed adequate image capture and displayed clear projections without artifacts or delays in transmission. The three-dimensional impression represented the most expensive part of the project, costing approximately US$ 800.00, an average amount that can be reduced by acquiring the printer for larger production. The costs of the additional components involved in the simulation protocol are as follows: mannequin preparation (US$ 40), camera (US$ 80), television and cables for image projection (US$ 380), guidewires (US$ 80), catheters (US$ 240), balloon catheter (US$ 300), and introducer sheath (US$ 80). The total cost of all materials was approximately US$ 2,000.

This study included 20 participants, comprising 15 medical students (novice group) and five surgeons (expert group). Regarding professional experience, all surgeons had at least three years of experience, and two of them (40%) had eight years of experience. Concerning medical students, 80% had been attending Medical School for at least three years, as shown in Table 1.

**Table 1:** Characteristics of novice and expert participants*.

	Novice group (n = 15)	Expert group (n = 5)
**Age, years**	23 ± 3	34 ± 2
**Previous contact with endovascular simulator**		
No	15 (100)	0 (0)
Yes	0 (0)	5 (100)
Complete years of endovascular practice**		
0–2	-	0 (0)
3–4	-	2 (40)
5–6	-	1 (20)
7 or more	-	2 (40)
**Current year in medical school** ^†^		
Second	3 (20)	-
Third	6 (40)	-
Fourth	4 (26.7)	-
Fifth	2 (13.3)	-
**Previous graduation** ^†^		
No	13 (86.7)	-
Yes	2 (13.3)	-
**Interest in surgical residency** ^†^		
No	0 (0)	-
Yes	15 (100)	-

* Categorical data are expressed as absolute counts and percentages in parentheses, and continuous data are expressed as mean ± standard deviation. χ^2^ or Fisher’s exact tests were used to compare categorical data. Student’s t-tests were used for all comparisons of continuous data;

** for expert group only;

† for novice group only.

Source: Elaborated by the authors.

All participants had prior experience with simulators in Medical School, like simulators for endotracheal intubation or cardiopulmonary resuscitation. However, only participants in the expert group (100%) had prior experience using endovascular simulators.

### Construct validity

When evaluating the time required to complete each exercise, exercise 1 demonstrated the shortest duration, likely reflecting its relative simplicity, whereas the remaining four exercises required substantially longer completion times (Table 2).

**Table 2:** Comparison of execution times between novice group and expert group*.

	Novice group (n = 15)	Expert group (n = 5)	Difference of means (s)	95%CI of difference (s)*	*p* -value
**Session 1 (seconds)**					
Exercise 1	44 ± 15.8	22.2 ± 6.3	21.8	6.3–37.3	0.008
Exercise 2	318.3 ± 104.2	64.6 ± 15.6	253.7	153.6–353.7	< 0.001
Exercise 3	339.9 ± 79.1	115.4 ± 29.7	224.5	147.3–301.6	< 0.001
Exercise 4	229.3 ± 134.8	37.8 ± 21.3	191.5	62.1–320.9	0.006
Exercise 5	269.9 ± 55.7	87 ± 24.6	182.9	128.2–237.7	< 0.001
**Session 2 (seconds)**					
Exercise 1	21.7 ± 7.7	16 ± 5.4	5.7	−2.1–13.6	0.143
Exercise 2	142 ± 43.8	54 ± 16.1	88.0	45.3–130.7	< 0.001
Exercise 3	222.7 ± 70.1	91.6 ± 21.5	131.1	63.1–199.1	0.001
Exercise 4	83.5 ± 31.1	45.2 ± 37	38.3	3.0–73.5	0.035
Exercise 5	190.8 ± 64.3	85.2 ± 16.4	105.6	43.5–167.7	0.002

* Data expressed as mean ± standard deviation. Student’s t test was used for all comparisons; 95%CI: 95% confidence interval.

Source: Elaborated by the authors.

All groups were then independently compared to assess their performance in each simulation training session. In session 1, a significant difference was observed in the time spent on all exercises, with the expert group achieving a significantly shorter average time to complete the tasks (*p* < 0.05) (Table 2).

In session 2, it was observed that for exercise 1, there was no significant difference between the two groups in completing the exercise (21.7 ± 7.7 *versus* 16 ± 5.4 seconds, 95% confidence interval -1.27–12.67, *p* > 0.05). However, for the other exercises (2, 3, 4 and 5), the expert group showed a significantly shorter average time than the novice group (*p* < 0.05) (Table 2).

Additionally, we evaluated whether there were differences in the time required for each type of exercise between the two sessions within each group. The novice group had a significant decrease in the time taken to perform all the five exercises in session 2 compared to the same exercises in session 1 (*p* < 0.001) (Table 3). In contrast, for the expert group, there was no significant reduction in the time spent on most exercises in session 2 compared to session 1 (*p* > 0.05), except for exercise 3, which presented a significantly reduced time in session 2 compared to session 1 (Table 3).

**Table 3:** Comparison of execution times between sessions in novice and expert groups*.

Execution time	Novice group (n = 15)		Difference of means (s)	95%CI of difference (s)*	*p* -value
	Session 1 (s)	Session 2 (s)			
Exercise 1	44 ± 15.8	21.7 ± 7.7	22.3	14.3–30.3	< 0.001
Exercise 2	318.3 ± 104.2	142 ± 43.8	176.3	129.1–223.5	< 0.001
Exercise 3	339.9 ± 79.1	222.7 ± 70.1	117.2	72.7–161.7	< 0.001
Exercise 4	229.3 ± 134.8	83.5 ± 31.1	145.8	73.8–218.0	0.001
Exercise 5	269.9 ± 55.7	190.8 ± 64.3	79.1	42.5–115.8	< 0.001
**Execution time**	**Expert group (n = 5)**		**Difference of means (s)**	95%CI of difference (s)*	***p* -value**
	**Session 1 (s)**	**Session 2 (s)**			
Exercise 1	22.2 ± 6.3	16 ± 5.4	6.2	-0.8–13.2	0.070
Exercise 2	64.6 ± 15.6	54 ± 16.1	10.6	-16.4–37.6	0.337
Exercise 3	115.4 ± 29.7	91.6 ± 21.5	23.8	8.8–38.8	0.012
Exercise 4	37.8 ± 21.3	45.2 ± 37	-7.4	-55.2–40.4	0.689
Exercise 5	87 ± 24.6	85.2 ± 16.4	1.8	-37.2–40.8	0.904

* Data expressed as mean ± standard deviation. The paired t-test was used for all comparisons.

Source: Elaborated by the authors.

**Figure 4:**
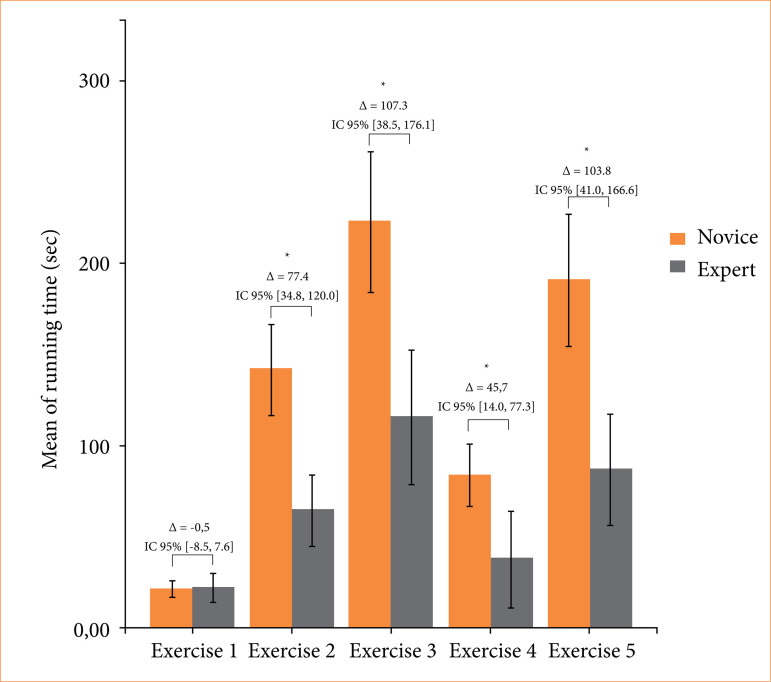
Comparison between the execution times of session 2 of the novice group and the times of session 1 of the expert group.

Comparing times in session 2 from novice group and the times observed in session 1 for expert group, it was noted that for the time spent on exercise 1, there were no significant differences between the two groups (21.7 ± 7.7 *versus* 22.2 ± 6.3 seconds, *p* = 0.904). However, when comparing the times for the other four exercises (exercises 2–5), the expert group consistently spent less time than the novice group (*p* < 0.05) (Fig. 4).

*p < 0.05 using Student’s test for all comparisons. Source: Elaborated by the authors.

### Face and content validity

Regarding the participants’ perceptions of the endovascular simulator activities, such as their performance, utility, and ease of use, the vast majority evaluated the simulator positively in various aspects, as shown in Table 4. Nearly all vascular surgeons considered the simulator suitable for training basic endovascular skills. In addition, all participants believed the device was useful for training novice residents.

**Table 4:** Content and face validity.

Training tool usefulness (content)*	Median score (range)
This simulator is suitable for training basic endovascular skills	5 (4–5)
My training during residency would have been optimized using this device	5 (4–5)
I performed all tasks without technical limitations related to the device’s structure	5 (5–5)
Time reserved to perform those exercises is compatible with most vascular training programs	5 (4–5)
I consider that skills improved using this simulator might be transferred to real life scenarios	5 (5–5)
**User realism (face)†**	
General configuration	8 (8–10)
Projected image	8 (8–10)
Exercises performed	10 (9–10)
Guidewires and catheters manipulation	10 (9–10)

* Likert scale with J score ranging from 1 to 5 (1 = strongly disagree; 2 = disagree; 3 = neutral; 4 = agree; 5 = strongly agree);

† rates ranging for 1 (worst) to 10 (best).

Source: Elaborated by the authors.

Finally, the expert group rated the simulator’s realism (face validity). The general configuration, projected image, instrument manipulation, and procedures performed were evaluated and represented as median scores. Table 4 presents the results of the questionnaire.

## Discussion

At the beginning of formal training, the expected initial effect is a reduction in total execution time, followed by subsequent refinements in technique during longitudinal follow-up^25^. In our study protocol, the time required to complete all five exercises demonstrated a statistically significant reduction in the novice group during the second cycle, reflecting a substantial improvement in performance following a brief period of simulator-based training. Specifically, for the first exercise, after the first cycle, the procedural time was comparable to that of experienced surgeons, which may represent a valuable finding in terms of surgical abilities acquisition.

Aeckersberg et al.^26^ reported that a low-fidelity virtual reality simulator for training beginners in basic tasks had a beneficial educational impact, making it a more affordable and accessible option. Regardless of the sophistication degree, it is recommended that these systems achieve excellence, reproducibility, and validation to be recognized as cost-effective, thereby guaranteeing their educational effectiveness^27,28^.

Norman et al^.29^ have previously compared learning from high-fidelity and low-fidelity simulations in many areas of medical knowledge. The authors concluded that the relationship between simulation fidelity and learning was neither unidimensional nor linear, resulting in potential performance improvement with no clear specific advantage to support one over another.

As expected, the group of vascular surgeons did not show a statistically significant difference in improvement between the first and second sessions for almost all stages. Because this simulator introduces basic endovascular skills, it was expected that the performance would vary slightly due to their high previous degree of experience. The statistically significant difference in exercise 3 may be related to several individual factors, such as motivation and focus on exercise execution, which could have been mitigated if the expert group had more volunteers. The sample size was defined pragmatically, based on feasibility and on ensuring completion of the study protocol. The small number of participants in the expert group (n = 5) limits the statistical power and the generalizability of the findings. These findings should be interpreted with prudence due to the risk of being prone to a type I error.

Relevant aspects of simulator design have been investigated to clarify whether technological advancements are directly associated with educational effectiveness^30^. From our perspective and in accordance with other authors^15,31^, low-cost simulators designed for psychomotor skill training are feasible and interesting strategies for basic learning. A much lower number of simulators in this category have been reported and validated compared to high-tech models based on virtual reality^16^.

Evaluations by endovascular surgeons showed excellent scores for facial and content validity. The limited number of expert evaluators, combined with the clustering of responses at the upper end of the scale, likely resulted in a ceiling effect, thereby reducing the discriminative capacity of the instrument. For this reason, these findings should be interpreted as preliminary and should not be overemphasized as robust psychometric evidence. The present study provides preliminary evidence of the simulator’s discriminative capacity across levels of expertise, as a clear difference was observed between expert users and novice participants.

Currently, transparent models are used by the industry for development, testing, and demonstration of new technologies, such as endografts and stents^22,32^. However, the literature needs to be more extensive regarding its role in endovascular education, and new tools using three-dimensional printing technology have been reported^33-36^. This method has been used to produce detailed models that increase the realism needed for high-fidelity simulations, as well as procedure-specific rehearsals of complex cases^37^. In a recent systematic review, only four endovascular simulators with three-dimensional models for interventional procedures were evaluated as educational tools^38^.

Detailed research on materials that can represent the characteristics of human vascular tissue is a field of enthusiasm^39^. Achieving realistic features is a challenge not only because of the investigation of new materials but also because of the high degree of complexity in the design of models and preparation for their use^40^.

Sinceri et al.^41^ previously reported a simulator using a transparent model produced by a numerical control laser machine with images projected from a static webcam, confirming its face and content validity. This study highlighted the remarkable features of realistic manipulation of endovascular instruments such as guidewires and catheters.

In addition to producing a three-dimensional-printed transparent arterial model, we added a system for projecting dynamic images from flexible support to improve realism and mimic fluoroscopic incidences. Because this simulator employs an optical system for image capture, ionizing radiation is avoided, thereby maintaining real-time visualization of wires and catheters. We believe this is one of the most relevant aspects of our study protocol.

This research had some limitations. Most importantly, this was a single-center study and not randomized. The number of participants was limited due to the low availability of volunteers. Since all students recruited were interested in pursuing a surgical career, this sample is not representative of the general student population, likely overestimating performance and involvement. Furthermore, we emphasize the need for additional randomized controlled trials comparing its usefulness with other teaching methods, such as virtual reality devices, which is important for future research to define the role of each approach in teaching endovascular procedures.

Additionally, this simulator still needs to be validated regarding its transferability to real-life situations. It is important to conduct more in-depth investigations to determine if the acquired skills can be transmitted to the surgical environment, especially if they can lead to better clinical results. Also, the durability of the simulator must be evaluated during long-term follow-ups. Quality criteria in conception and design should go far beyond physical similarity, leading to behavioral changes and improvements in procedural performance^42^.

Including an intermediate group of residents’ experiences was initially planned, but owing to their limited number in our institution, this would lead to additional bias. Further investigations should evaluate the results of longer training periods for novice volunteers and include other metrics. The time registered for completing each task represents a primary quantitative parameter. More detailed performance assessments can adapt the Objective Structured Assessment of Technical Skills^43,44^ scores for those endovascular procedures. The participation of undergraduate students in this research should be understood mainly as part of an initial validation strategy rather than as evidence supporting broad implementation of endovascular simulation within the undergraduate medical curriculum.

There are some points for potential future improvements in our simulator. Building a camera support that resembles a C-arm and developing software that converts images from the camera into a fluoroscopic pattern may add more realism. Furthermore, an ultrasonography-guided arterial puncture model can be adapted to the femoral site. This system, based on a gelatin framework, has been previously described to mimic the acoustic properties of human tissues^45^. Our team did not include this in the experimental testing protocol because this set of skills diverged from the focus of this study. Finally, the expert group did not mention the absence of water inside the arterial model as a disadvantage for performing tasks. Nevertheless, a hydraulic system with a transparent viscous solution simulating blood properties can be integrated to generate a pulsatile flow.

Three-dimensional-printed synthetic components that replicate the human vascular system must be improved^19^. The currently available models are still incapable of completely reproducing functional characteristics such as elasticity, deformability, and rigidity. The identical curves and variations in thickness presented by actual aortas in computed tomography scans could not be reproduced exactly by the three-dimensional printer employed because transparency would be compromised. Mimicking those aspects are critical because they directly influence the interaction between the instruments and the addressed vascular bed, thus predicting the technical difficulties that may be encountered in real cases. Pathological conditions such as calcified atherosclerotic plaques or dissections are difficult to represent using current three-dimensional-printed models.

## Conclusion

The construction of a low-fidelity simulator for training basic endovascular skills is feasible, and its use in training demonstrates improvements in the execution of simulated tasks specially for undergraduate medical students. Our results provided a positive evaluation of a preliminary device that offers a radiation-free environment and utilizes a camera-assisted approach for its operation. Further studies are required to compare its beneficial impact with other current methods and the transferability of skills to real-life situations.

## Data Availability

All data sets were generated or analyzed in the current study.
